# Comparing mouse and human brains

**DOI:** 10.7554/eLife.90017

**Published:** 2023-07-10

**Authors:** Hovy Ho-Wai Wong, Christina You Chien Chou, Alanna Jean Watt, Per Jesper Sjöström

**Affiliations:** 1 Centre for Research in Neuroscience, Department of Medicine, The Research Institute of the McGill University Health Centre Montreal Canada; 2 Integrated Program in Neuroscience, McGill University Montreal Canada; 3 Department of Biology, McGill University Montreal Canada

**Keywords:** cerebral cortex, synaptic connections, neural circuits, Human

## Abstract

Inhibitory circuit motifs in the mouse brain and the human brain are strikingly similar.

**Related research article** Kim MH, Radaelli C, Thomsen ER, Monet D, Chartrand T, Jorstad NL, Mahoney JT, Taormina MJ, Long B, Baker K, Bakken TE, Campagnola L, Casper T, Clark M, Dee N, D’Orazi F, Gamlin C, Kalmbach BE, Kebede S, Lee BR, Ng L, Trinh J, Cobbs C, Gwinn RP, Keene CD, Ko AL, Ojemann JG, Silbergeld DL, Sorensen S, Berg J, Smith KA, Nicovich PR, Jarsky T, Zeng H, Ting JT, Levi BP, Lein E. 2023. Target cell-specific synaptic dynamics of excitatory to inhibitory neuron connections in supragranular layers of human neocortex. *eLife* **12**:e81863. doi: 10.7554/eLife.81863.

The mouse is used as a model organism in many areas of research, including neuroscience. The human brain is clearly larger than the mouse brain, and it is also more complex, but how similar are they at the level of individual neuron types and their connections (Figure 1A)?

**Figure 1.**
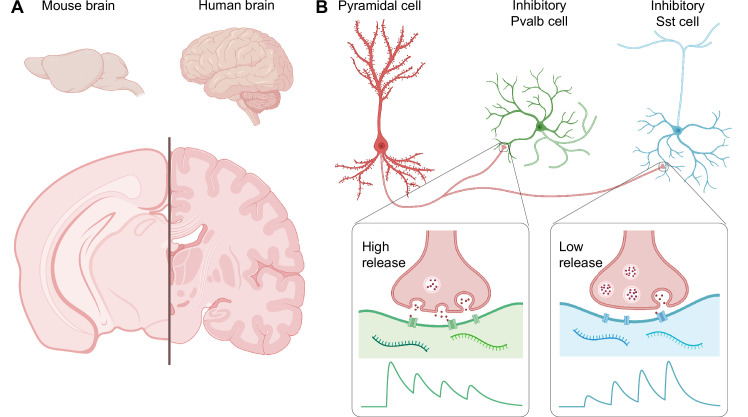
Comparing the mouse brain and the human brain. (**A**) The human brain is larger than the mouse brain, and also much more complex, with more neocortical invaginations and a greater number of specialized areas. (**B**) However, at the level of individual neuron types and their connections, mouse and human brains are similar. For example, Kim et al. found in the human brain a circuit motif that includes an excitatory neuron called a pyramidal cell, and an inhibitory interneuron – either a Pvalb cell or a Sst cell. The rapid and high release of neurotransmitters at the synapse between the pyramidal cell and the Pvalb cell leads to early-onset inhibition (green trace), while the low and gradually ramped-up release of neurotransmitters at the synapse with the Sst cell leads to late-onset inhibition (blue trace). Since the mouse and human brain share these circuit motifs, the mouse brain may serve as a good model of the human brain.

Figure created using BioRender.

Some argue that we make too much of findings in mice. For instance, the twitter account @justsaysinmice retweets eye-catching scientific claims with the comment “IN MICE” (Piper, 2019). Indeed, a number of electrophysiology studies have compared mouse and human brain circuits, and many of these studies have revealed key differences (Mansvelder et al., 2019). However, it could be argued that looking for differences guarantees that they will eventually be found, even though their significance might not be clear. An alternative tactic is to explore similarities between the mouse brain and the human brain (Szegedi et al., 2020).

At the level of individual neuron types and their connections, the brain is made up of repeated building blocks known as circuit motifs that contain combinations of interconnected excitatory and inhibitory neurons. A number of studies in mouse models of autism and epilepsy have found that these conditions are associated with a lack of balance between excitation and inhibition in the brain (Nelson and Valakh, 2015). Two key types of inhibitory interneurons have been well studied in mice: the parvalbumin (Pvalb) cells, which silence target neurons relatively quickly, and the somatostatin (Sst) cells, which take longer to act (Figure 1B; Blackman et al., 2013). Then again, is this just in mice, or are motifs with Pvalb or Sst cells also found in humans?

Now, in eLife, Mean-Hwan Kim and colleagues – who are based at the Allen Institute for Brain Science, the University of Washington, and the Swedish Neuroscience Institute – report that inhibitory circuit motifs in humans and mice are strikingly similar (Kim et al., 2023). Building on recent work in which they used high-throughput transcriptomic profiling (Bakken et al., 2021), the researchers compared the transcriptomes of cells from the mouse and human cortex. This revealed over 70 genes that were differentially enriched in Pvalb and Sst cells. Many of these genes were related to the connections between neurons, suggesting that they determine the properties of the synapses of these two cell types. The similar cell-type-specific genetics seen in mice and humans suggest that these interneuron subclasses are evolutionarily conserved.

To explore this idea, Kim et al. obtained human cortical tissue samples from neurosurgical resections. To make the most of these precious samples, some brain slices were used acutely, meaning right away, whereas others were kept for days as a cultured preparation. The acute slices may represent the intact brain better, but the cultured slices can be studied with a wider range of techniques. For instance, the interneurons in the cultured samples can be genetically labelled for easy identification. When the results from the acute and cultured slices were compared, there were no appreciable differences, thus validating the use of cultured slices.

Kim et al. used a combination of different techniques – multiple patch-clamp recording, cell morphology reconstruction, and multiplexed fluorescent in-situ hybridization (mFISH) – to study the slices. They found two types of inhibitory circuit motif that worked in the same way in both mice and humans. The synaptic dynamics of the motif formed by an excitatory neuron called a pyramidal cell and a Pvalb cell promoted early early-onset inhibition, whereas that formed with a Sst cell favored late-onset inhibition (Figure 1B; Blackman et al., 2013). These findings argue that human and mouse brains are comprised of similar inhibitory circuit motifs.

Two factors make it challenging to study the human brain – it is difficult to obtain human brain tissue, and most experimental techniques have low throughput. For example, the mFISH procedure used for identifying patched cells was both slow and prone to failure. Kim et al. overcame this problem by using machine learning to rapidly classify cell types using only electrophysiology data, and they were able to identify Pvalb cells with ~76% accuracy. In the future, it should be possible to increase throughput even more by also using recently developed optogenetic approaches to circuit mapping (Hage et al., 2022).

It should also be noted that the human tissue samples used by Kim et al. might be pathological because they came from patients with epilepsy or brain tumors. However, their similarity to healthy rodent tissue suggests that these human samples were not aberrant but representative.

In summary, Kim et al. provide compelling evidence that inhibitory circuit motifs are conserved across mice and humans. This has far-reaching implications, as it argues that the knowledge generated from decades of rodent research is relevant to human neurophysiology. In addition, the methods they developed to increase experimental throughput in this study will help researchers to learn more from precious samples of human brain tissue in the future.

There are many other types of inhibitory interneurons beyond the Pvalb and Sst cells discussed here (Gouwens et al., 2020). Moreover, our understanding of these interneurons and the circuit motifs they form is limited, as is our knowledge of their role in disease (McFarlan et al., 2023). It is therefore reassuring to know that future neuroscience research in mice will continue to reveal secrets pertinent to the human brain.
